# Functional Involvement of circRNAs in the Innate Immune Responses to Viral Infection

**DOI:** 10.3390/v15081697

**Published:** 2023-08-05

**Authors:** Mohamed Maarouf, Lulu Wang, Yiming Wang, Kul Raj Rai, Yuhai Chen, Min Fang, Ji-Long Chen

**Affiliations:** 1 Key Laboratory of Animal Pathogen Infection and Immunology of Fujian Province, College of Animal Sciences, Fujian Agriculture and Forestry University, Fuzhou 350002, China; mohamed_maarof@vet.suez.edu.eg (M.M.); m18539755203@163.com (L.W.); zqpjy2178@gmail.com (Y.W.); kulrajrai701@gmail.com (K.R.R.); chenyuhai@im.ac.cn (Y.C.); 2 CAS Key Laboratory of Pathogenic Microbiology and Immunology, Institute of Microbiology, Chinese Academy of Sciences (CAS), Beijing 100101, China; fangm@im.ac.cn; 3 Department of Virology, Faculty of Veterinary Medicine, Suez Canal University, Ismailia 41522, Egypt; 4 Fujian Province Joint Laboratory of Animal Pathogen Prevention and Control of the “Belt and Road”, College of Animal Sciences, Fujian Agriculture and Forestry University, Fuzhou 350002, China; 5 Department of Microbiology, ShiGan International College of Science and Technology/ShiGan Health Foundation, Narayangopal Chowk, Kathmandu 44600, Nepal

**Keywords:** circRNAs, innate immunity, virus infection, virus–host interaction, circRNA vaccine

## Abstract

Effective viral clearance requires fine-tuned immune responses to minimize undesirable inflammatory responses. Circular RNAs (circRNAs) are a class of non-coding RNAs that are abundant and highly stable, formed by backsplicing pre-mRNAs, and expressed ubiquitously in eukaryotic cells, emerging as critical regulators of a plethora of signaling pathways. Recent progress in high-throughput sequencing has enabled a better understanding of the physiological and pathophysiological functions of circRNAs, overcoming the obstacle of the sequence overlap between circRNAs and their linear cognate mRNAs. Some viruses also encode circRNAs implicated in viral replication or disease progression. There is increasing evidence that viral infections dysregulate circRNA expression and that the altered expression of circRNAs is critical in regulating viral infection and replication. circRNAs were shown to regulate gene expression via microRNA and protein sponging or via encoding small polypeptides. Recent studies have also highlighted the potential role of circRNAs as promising diagnostic and prognostic biomarkers, RNA vaccines and antiviral therapy candidates due to their higher stability and lower immunogenicity. This review presents an up-to-date summary of the mechanistic involvement of circRNAs in innate immunity against viral infections, the current understanding of their regulatory roles, and the suggested applications.

## 1. Introduction

The human genome project showed that less than 2% of the human genome comprises protein-coding genes [1]. Nevertheless, most genomic DNA is a transcription template, indicating that the human transcriptome predominantly contains non-coding RNAs (ncRNAs) [2,3]. ncRNAs have been demonstrated to play crucial roles in the regulation of gene expression by impacting target genes’ transcription or post-transcriptional modifications. ncRNAs can be divided into two major categories: small ncRNAs (sncRNAs) and long ncRNAs (lncRNAs) [4]. sncRNAs are shorter than 200 nucleotides (nt) in length, and these include piwi-interacting RNAs, microRNAs (miRNAs), transcription initiation RNAs, and endogenous small interfering RNAs [5]. LncRNAs are longer than 200 nt in length and constitute most of the non-coding transcriptome in mammals [6,7].

Circular RNAs (circRNAs) were initially discovered in RNA viruses such as the Sendai virus, hepatitis D virus (HDV), and plant viroids in the 1970s [8,9,10,11]. Nevertheless, circRNAs were thought to be viral genomes or the results of pre-mRNA alternative splicing. Hence, they received little attention in the past [12]. Current advances in high-throughput sequencing technologies have enabled scientists to undertake comprehensive investigations of the structure, expression profile, and functions of circRNAs, as well as the mechanisms underlying their roles [13]. CircRNAs have been discovered in a wide range of plant and animal species [14]. Some circRNAs are evolutionarily conserved across related species [15]. The extensive presence of circRNAs in eukaryotic cells indicates that circRNAs are not only unintended products of RNA splicing events but rather an essential component of the ncRNA family [16].

CircRNAs are now established ncRNA family members formed via backsplicing introns, exons, or both [17]. CircRNAs are distinguished by their closed circular structure, and lack of 5′ N7-methylguanosine (m7G) cap ends or 3′ Poly (A) tails [18]. CircRNAs are a highly stable ncRNA form because they are naturally resistant to RNA nucleases [19]. Recently, the physiological and pathological roles of circRNAs have drawn researchers’ attention, as an increasing number of data demonstrate that circRNAs can serve various functions through multiple mechanisms [20]. It has been shown that circRNAs play numerous roles in cellular processes, such as the modulation of gene expression and alternative splicing, sponging to miRNAs or proteins, providing translation templates, rRNA and tRNA synthesis modulators, and so on [21]. Remarkably, many circRNAs exhibit altered expression levels in response to specific disease conditions or infections with pathogens, implying a link between circRNAs and the emergence and progression of human and animal disorders [22].

Interestingly, despite the significant focus on the study of the relationship between circRNAs and cancer, multiple reports have also suggested the involvement of circRNAs in innate immunity against viral infection. Typically, viral infection dysregulates the expression of circRNAs, which, in turn, could regulate viral replication by modulating innate immunity, providing novel insights into the diagnosis and treatment of viral infectious diseases. Here, in this review, we will discuss the taxonomy, biosynthesis, functions, and mechanism underlying the action of circRNAs and highlight their relevance to antiviral immunity and potential applications, such as antiviral therapeutics, vaccine candidates, and diagnostic and prognostic biomarkers.

## 2. Physical and Chemical Characteristics of Circular RNAs

### 2.1. RNA Circularization

Unlike conventional linear splicing, which forms a linear 5′ to 3′ mRNA of joined exons, circRNAs feature a covalently closed structure lacking the 5′-cap and 3′-poly(A) tail [23]. The conventional splicing patterns of circRNAs, known as exon-skipping and backsplicing [17], have been observed both in vivo and in vitro. Nevertheless, evidence suggests that backsplicing is more significant, as this pattern is widely reported [16].

Based on origin and composition, circRNAs may be categorized into three categories: circular intronic RNAs (ciRNAs), exonic circRNAs (ecircRNAs), or exon-intron circRNAs (eiciRNAs) [19]. EcircRNAs, composed of one or multiple exons, constitute more than 80% of the detectable circRNAs and are preferentially generated by intron pairing-driven circularization or lariat-driven circularization [24,25]. The flanking introns adjacent to the backspliced exons are crucial for circRNA biogenesis, as they contain complementary sequences that base-pair and form hairpin-like structures, allowing the 5′ and 3′ splicing sites to be closer for circularization to occur [26]. These sequences may consist of Alu repeats of ~300 nt in length or non-repetitive elements [26]. Alu repeats-driven circularization is often complex as the inverted Alu repeats may pair across introns and induce different exon circularization events; consequently, one single gene locus can produce various circRNAs [18,26].

EiciRNAs utilize an exon-skipping strategy for circularization, and contain flanking intron sequences on the exonic core sequence’s off-side [27]. CiRNAs are generated via a lariat-derived process that requires a consensus GU-rich domain close to the 5′-end splicing site and a C-rich domain close to the breakpoint, and then the uncircularized intron sequences are then sequestered [23]. CircRNAs are predominantly cytoplasmic, but ciRNAs and eiciRNAs are exclusively nuclear, suggesting roles in nuclear processes, such as transcriptional regulation [28].

Since circRNAs and their cognate mRNA share the same pre-mRNA and most splicing sites, circRNA backsplicing and mRNA splicing often compete for transcription against each other [8,29]. Nevertheless, due to unfavorable spliceosome assembly at backsplicing sites, the efficiency of backsplicing is significantly lower than that of canonical splicing [30].

### 2.2. circRNAs versus mRNAs

circRNAs exhibit the same nucleotide sequence as their corresponding linear RNAs, except for the back-splicing junction (BSJ) site. Thus, distinguishing the expression of a particular circRNA from its linear cognate has been challenging on multiple scales, including identification, validation, and loss- and gain-of-function investigations [19]. Nevertheless, the BSJ site enables the utilization of divergent primers to detect specific circRNAs while avoiding the undesired signal of their cognate linear mRNAs [31].

In addition, most circRNAs are stable, with half-lives ranging between 18.8 and 23.7 h [30,32], which is much longer than the range of 4.0–7.4 h that their linear RNA cognates have [32]. This stability is presumably a result of their resistance to linear RNA degradation machinery. Consequently, in slowly dividing or nondividing cells and tissues, certain circRNAs could accumulate to high levels [19,33]. However, it is suggested that following viral infection or poly(I:C) stimulation, the RNase L could degrade the transcribed circRNAs, a mechanism necessary for early innate immune responses [34]. Notably, after being transcribed, circRNAs could undergo N6-methyladenosine (m^6^A) modifications [35]. m^6^A-circRNAs can then be recognized by YTHDF2 and HRSP12, resulting in circRNA degradation by the RNAse P/MRP ribonuclease complex [36]. Some circRNAs are degraded after perfect sponging to miRNA via the AGO2-mediated cleavage of circRNA [37]. Additionally, another degradation mechanism is that of the G3BP1 endonuclease, as it complexes with the RNA-binding protein UPF1 and decays highly structured circRNAs [38].

Another functionally important distinction between circRNAs and their cognate mRNAs is immunogenicity [39,40,41]. Whereas self-circRNAs are not immunogenic, the RNA pattern recognition receptors, Toll-like receptor 7/8 and retinoic-acid-inducible gene-I (RIG-I), may be activated by exogenous circRNAs, making the circRNA itself immunostimulatory [41]. The immunogenicity of circRNA could be unfavorable to the circRNAs’ translation efficiency, their half-life stability, and biomedical applications. It has been demonstrated that impure circRNA formulas elicit potent immunological responses in cells [42,43,44].

### 2.3. Mechanisms Underlying Action of circRNAs

Studies have established that circRNAs play crucial roles in the modulation of physiological processes and the progression of numerous diseases [45]. In the past few years, the mechanisms underlying the action of circRNAs, such as the regulation of gene expression, microRNA reservoirs, and microRNA sponges, the ability to encode proteins, etc., have been progressively reported [19].

MicroRNA response elements (MREs) are miRNA-complementary sequences in the 3′-UTRs of target mRNAs [46]. Similarly, circRNAs, primarily cytoplasmic, include several MREs that may bind to target miRNAs, thereby functioning as competitive endogenous RNAs (ceRNAs) [47]. Additionally, lacking the 5′-cap or 3′-poly(A) tail, circRNAs are resistant to RNase degradation, enabling them to serve as potent sponges for miRNA [48]. On the other hand, some circRNAs possess the capacity to stabilize or stimulate the actions of miRNAs, a property known as miRNA reservoirs [49].

Moreover, circRNAs can bind to specific proteins called RNA-binding proteins (RBPs), which serve as protein sponges [19]. RBPs exhibit complementary sequences to bind with their target RNA, and circRNAs can modulate protein activity by docking to the active sites of RBPs [50].

Of note is that in 2015, circRNAs were reported in fruit flies to encode translatable proteins and peptides [51]. This discovery established the theoretical foundation that paved the way for circRNA vaccine development [52]. Multiple reports have demonstrated that, unlike conventional mRNAs, circRNAs initiate translation not at the 5′ cap but rather at the m^6^A-induced ribosome engagement site or the internal ribosomal entry site (IRES) [53,54,55].

## 3. Involvement of circRNAs in Innate Immunity against Viral Infection

**Figure 1:**
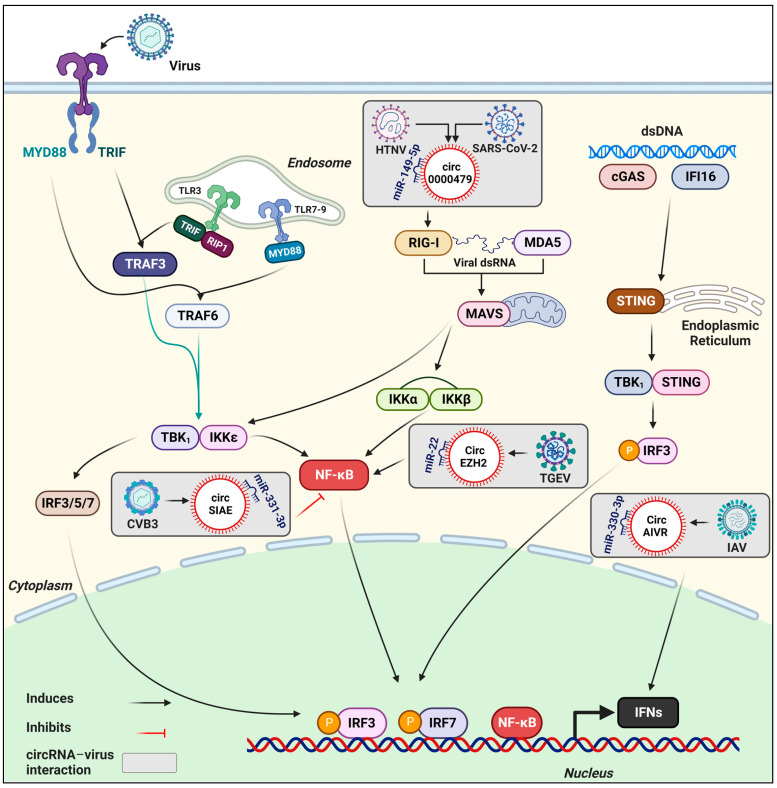
Mechanistic involvement of some circRNAs in innate immunity against some representative viral infections. As reviewed by Rai et al. [57], virus detection by PRRs triggers innate immune signaling via the activation of specific adaptor proteins (MYD88, MAVS, TRIF, STING, etc.) to subsequently activate other transcriptional factors, including NF-κB, IRF3/5/7, and others. The translocation of activated transcriptional factors into the nucleus induces the expression of IFNs. The mechanistic involvement of several circRNAs in the modulation of immune responses has been revealed. For instance, circ_0000479 sponged miR-149-5p and regulated RIG-I expression, thus impacting HTNV and SARS-CoV2 viral replication [63,64]. CircRNA AIVR inhibited IAV replication by predominantly absorbing miR-330-3p and enhancing CREBBP expression, thus facilitating the production of IFN-β [59]. CircSIAE suppressed CVB3 replication by targeting miR-331-3p and TAOK2, and impacting the levels of p-NF-κB [65]. CircEZH2 promoted the activation of NF-κB via sponging miR-22 after TGEV infection [66,67]. HTNV: Hantaan virus; HBV: Hepatitis B virus; TGEV: Transmissible Gastroenteritis Virus; IAV: Influenza A virus; CVB3: Coxsackievirus B3. Created with BioRender.com (accessed on 4 August 2023).

Innate immunity provides the first line of defense against infections with pathogens [56,57], and it possesses a crucial function in virus recognition and the subsequent activation of the adaptive immune response. Pathogen-associated molecular patterns (PAMPs) are viral components that can be sensed by the innate immune molecules’ pathogen recognition receptors (PRRs). PAMPs include, for instance, viral double-stranded RNA, viral single-stranded RNA, and viral DNA. PPRs, such as RIG-I, Toll-like receptors, and nucleotide oligomerization domain (NOD)-like receptors, are indispensable for the activation of innate immune signaling that ultimately induces the production of various cytokines and antiviral molecules, including interferons (IFNs) [56,58] (Figure 1).

Interestingly, recent studies have demonstrated the mechanistic involvement of circRNAs in viral infections, the regulation of innate immune responses, and other biological processes of antiviral immunity [31,59]. Numerous virus- and host-derived circRNAs are shown to regulate antiviral immune responses [21,60,61,62].

### 3.1. Host-Coded circRNAs Involved in Immune Responses

The dysregulation of cellular gene expression occurs in responses to multiple stimuli, including infections [68]. Among them, viral infections have been reported to induce a differential expression of host circRNAs, potentially enhancing or inhibiting the innate immune response or impacting viral pathogenesis. Although the precise mechanisms underlying the involvement of circRNAs in antiviral innate and adaptive immune responses and viral pathogenesis are elusive, several studies have revealed their significance. For example, multiple reports have revealed various differentially expressed host circRNAs and established a circRNA–miRNA–mRNA regulatory network in immune responses to infections with multiple viruses, including Hantaan Virus, Human Immunodeficiency Virus, Coxsackievirus B5, Coxsackievirus A16, Rabies Virus, Peste-Des-Petits-Ruminants Virus, Japanese Encephalitis Virus, Porcine Endemic Diarrhea Virus, Pseudorabies Virus Type II, Foot-And-Mouth Disease Virus, Human Papillomavirus E7, Avian Leukemia, Influenza Virus, Infectious Bursal Disease Virus, Enterovirus A71, Swine Hepatitis E Virus, Or Middle East Respiratory Syndrome Coronavirus, SARS-CoV-2 virus, and so on [63,69,70,71,72,73,74,75,76,77,78,79,80,81,82,83,84,85,86,87]. In addition, plant viruses are also reported to alter circRNAs expression. For instance, Maize Iranian mosaic virus (MIMV) induced the dysregulation of circRNAs expression in MIMV-infected maize [88]. Nevertheless, the precise functions of the altered circRNAs during viral infections are still the subject of ongoing research.

Various circRNAs have been implicated in host–virus interactions. Here, we present a brief overview of the most up-to-date comprehension of the roles of circRNAs in modulating immune responses and impacting the pathogenesis of viruses with medical or veterinary importance (Table 1 and Figure 1).

#### 3.1.1. Hepatitis B Virus (HBV)

HBV is an exclusively hepatotropic virus that can cause persistent infections (chronic hepatitis B (CHB)) and, in extreme cases, cirrhosis and hepatocellular cancer (HCC). It was shown that hsa_circ_0004812 was highly upregulated in HCC tissues from CHB patients. The robust expression of hsa_circ_0004812 enhanced HBV-induced immunosuppression by sponging to miR-1287-5p, whereas knocking down hsa_circ_0004812 promoted interferon (IFN)-α/β production and suppressed viral propagation and multiplication, suggesting a putative HBV therapeutic target [90].

It was also found that circ-ATP5H was upregulated in HCC-HBV-infected tissues. Circ-ATP5H knockdown impacted HBV replication through sponging miR-138-5p. Circ-ATP5H modulates TNFAIP3 by binding to miR-138-5p. Interestingly, circ-ATP5H boosted HBV multiplication by altering the miR-138-5p/TNFAIP3 axis, revealing a potential novel biomarker for HBV-positive HCC therapy [92].

In addition, HCC tissues, HepG2, and Huh7 cell lines showed elevated levels of circBACH1 and MAP3K2 and diminished levels of miR-200a-3p. CircBACH1 depletion or miR-200a-3p overexpression significantly impaired HBV replication. Studies revealed that circBACH1 governs HBV replication via the miR-200a-3p/MAP3K2 pathway [91].

#### 3.1.2. Influenza Virus

Influenza is a contagious respiratory disease caused by the influenza A (IAV) and B viruses. Yu et al. [95] reported that following IAV H1N1 infection, A549 cells showed a dramatic increase in circ-GATAD2A expression. It was observed that circ-GATAD2A overexpression facilitated H1N1 replication via the inhibition of VPS34-dependent autophagy, whereas circ-GATAD2A silencing reduced H1N1 titers [95]. It was also reported that circRNA_0050463 sponged to miR-33b-5p, therefore promoting the expression of eukaryotic translation elongation factor 1 alpha 1 and boosting the replication of IAV [94]. In addition, circRNA AIVR was demonstrated to inhibit IAV multiplication principally by sponging to miR-330-3p, inhibiting its binding to the mRNA of the CREB-binding protein, thereby accelerating IFN production (Figure 1) [59].

Recently, circMertk was reported by Qiu et al. [31] as a novel circRNA derived from pre-MerTK. Interestingly, the overexpression or silencing of circMerTK enhanced or inhibited the replication of the IAV and Sendai viruses, respectively. CircMerTK silencing stimulated the secretion of type I IFNs and the expression of interferon-stimulating genes, while the robust expression of circMerTK impaired their expression at both mRNA and protein levels [31]. Additionally, in another report, RNA-seq of A549 cells in response to avian influenza (AIV) or IAV infections led to the identification of multiple sets of altered circRNAs’ expression, and the authors selected six circRNAs (hsa_circ_0005870, hsa_circ_0006104, hsa_circ_0009609, hsa_circ_0060300, hsa_circ_0009365, and hsa_circ_0003428) for further analysis [96]. The authors suggested that the selected circRNAs may influence the cell cycle process and the endocytosis pathway via an in silico-established ceRNA network [96].

#### 3.1.3. Middle East Respiratory Syndrome Coronavirus (MERS-CoV)

MERS-CoV is an extremely pathogenic zoonotic virus that was first reported in humans in Saudi Arabia and Jordan in 2012, with a high mortality rate and unpredictable incidence [100]. Interestingly, Calu-3 cells infected with MERS-CoV showed elevated circFNDC3B and circCNOT expression levels. Further studies showed that silencing these particular circRNAs decreased the cellular viral load and indirectly influenced MAPK signaling pathways [69]. Additionally, another study observed that the inhibition of hsa_circ_0004445 significantly reduced MERS-CoV replication by 50–75% via binding to hnRNP C [93].

#### 3.1.4. Hepatitis C Virus (HCV)

HCV, a member of the Flaviviridae family, is the causative agent of hepatitis C, a worldwide health concern with an estimated 7.1 million people chronically infected with HCV [101]. HCV viral infectivity was significantly diminished after the depletion of circTIAL and circEXOSC in HCV-infected cells [97]. Additionally, the upregulation of circPSD3 in response to hepatitis C virus infection was reported to impact HCV viral abundances significantly, thus acting as a proviral factor in the post-translational regulation of HCV RNA amplification [97].

#### 3.1.5. Transmissible Gastroenteritis Coronavirus (TGEV)

Transmissible gastroenteritis is an infectious disease in swine, particularly piglets, characterized by severe vomiting and diarrhea caused by TGEV [66]. CircEZH2 was found to be downregulated after TGEV infection. CircEZH2 promoted NF-κB activation by targeting miR-22 in intestinal porcine enterocyte (IPEC-J2) cells (Figure 1). The mitochondrial permeability transmission pore (mPTP) opening of IPEC-J2 was induced by TGEV infection and repressed by miR-22 sponging to circEZH2. Interleukin 6 (IL-6) and hexokinase 2 (HK2) were identified as miR-22 targets. TGEV-induced mPTP opening might be controlled by two pathways: the circEZH2/miR-22/IL-6/NF-κB axis and the circEZH2/miR-22/HK2 axis [66,67].

#### 3.1.6. Ebola Virus (EBOV)

EBOV causes severe and typically fatal Ebola virus disease (EVD) [102]. Wang et al. [103] demonstrated that circ-chr19 boosted the claudin-18 (CLDN18) expression by sponging miR-30b-3p, thereby serving as a ceRNA during EBOV infection [103]. Since CLDN18 regulates cellular permeability, the basis of EBOV pathogenesis, circ-chr19 could be a promising target for EVD therapy.

#### 3.1.7. Human Immunodeficiency Virus (HIV)

Over 75 million individuals have contracted HIV worldwide. Untreated HIV infections are associated with progressive CD4+ T cell depletion and numerous immunological abnormalities [104]. Interestingly, the expression pattern and function of circRNAs in the pathophysiology of HIV were analyzed in peripheral blood mononuclear cells obtained from early HIV-infected patients [87]. The authors identified 15,145 unique circRNA transcripts, and the circRNA–miRNA–mRNA network uncovered that dysregulated circRNAs contributed to HIV-1 multiplication by regulating the expression of CCNK, CDKN1A, and IL-15 genes. Previous studies proved that circRNAs played a role in HIV replication and indicated their potential therapeutic application [87].

#### 3.1.8. Severe Acute Respiratory Syndrome Coronavirus 2 (SARS-CoV-2)

SARS-CoV-2 is a highly pathogenic and contagious coronavirus that evolved in 2019 and resulted in a global pandemic of acute respiratory illness [52]. A recent study by Firoozi et al. [64] has suggested that hsa_circ_0000479, induced by SARS-CoV-2, may regulate the immune response to SARS-CoV-2 by binding to hsa-miR-149-5p and influencing the expression of IL-6 and RIG-I (Figure 1) [64].

#### 3.1.9. Kaposi Sarcoma-Associated Herpesvirus (KSHV)

KSHV, or Human Herpesvirus-8, is the causative agent of Kaposi sarcoma [105]. According to several reports, KSHV, Epstein–Barr virus (EBV), and human cytomegalovirus induced hsa_circ_0001400 expression. The upregulation of hsa_circ_0001400 promoted the expression of tumor necrosis factor-alpha and diminished the production of two viral genes (replication and transcription activator and latency-associated nuclear antigen), which govern latent and lytic infection, respectively [89]. hsa_circ_0001400 promoted the cell cycle, suppressed apoptosis, and activated multiple immune genes’ expression (TAP2, ICAM1, CD40, etc.). hsa_circ_0001400’s capacity to impact viral genes implies that it may exhibit potential antiviral properties, given that numerous viruses trigger circ_0001400 expression, substantiating its importance [106].

#### 3.1.10. Herpes Simplex Virus Type 1 (HSV-1)

HSV-1 is a herpesvirus causing vesicular eruptions, most commonly in the orolabial and genital mucosa [107]. The analysis of top dysregulated circRNAs and their circRNA-miRNA–mRNA regulatory axis after HSV-1 infection (circRNA14189, circRNA14556, circRNA15053, and circRNA15655) revealed that a substantial number of genes related to immunity in the NOD-like receptor/JAK-STAT signaling pathways could be governed by HSV-1-induced circRNAs [107].

#### 3.1.11. Non-Mammalian Viruses

The majority of the studies on circRNAs focus on mammalian circRNAs, but numerous reports have addressed that non-mammalian species can also encode circRNAs that are implicated in immunity against viral infections. For instance, in teleost fish infected with *Siniperca chuatsi* rhabdovirus, circBCL2L1 was found to be significantly upregulated [108]. CircBCL2L1 could act as a ceRNA, enhancing the innate immune response by sponging to miR-30c-3-3p and influencing TRAF6, thereby inducing NF-κB/IRF3-mediated innate immunity and inflammatory pathways [108].

### 3.2. Virus-Coded circRNAs Affecting Innate Immunity

Circular RNAs were first discovered in a small number of viruses in the 1970s [11]. Since then, multiple DNA and RNA viruses have been shown to encode viral circRNAs [109,110,111,112], and several reports have investigated the crucial role of virus-coded circRNAs in the intricate virus–host interaction. Nonetheless, the precise biological roles of virus-coded circRNAs are still elusive [113,114].

For instance, circBART2.2, an EBV-encoded circRNA, was reported to be significantly upregulated in nasopharyngeal carcinoma (NPC), where it upregulates programmed death-ligand 1 (PD-L1) expression levels and inhibits T-cell functions in vivo and in vitro [115]. circBART2.2 assisted in immune escape by binding to the RIG-I helicase domain and activating transcription factors NF-κB and IRF3, resulting in increased PD-L1 transcription and the inhibition of the activation of the effector T lymphocytes [115].

In addition, it has been recognized that circRNAs generated by SARS-CoV-1, SARS-CoV-2, and MERS-CoV induce the expression of genes linked to mRNA processing and splicing during the initial stages of viral infection. In contrast, late-stage circRNAs modulate genes implicated in metabolism, autophagy, cancer, and viral infection [112].

It has also been observed that Merkel cell polyomavirus (MCV) expresses multiple circRNAs [116]. Of note, is that circMCV-T, the most highly expressed MCV-circRNA, modulated MCV replication by sponging to the miR-M1-loaded RISC complex, thus stabilizing the transcripts of linear T-Ag, and enhancing the expression of T-Ag. The depletion of circMCV-T was accompanied by the degradation of T-Ag linear transcripts via the miR-M1-induced RISC complex, inhibiting T-Ag production and, consequently, affecting MCV replication [116].

Additionally, some non-mammalian viruses have been shown to encode circRNAs. For example, *Bombyx mori* cypovirus (BmCPV) is an RNA virus affecting silkworms that can cause developmental retardation and severe economic losses [117]. BmCPV was reported to encode circRNA-vSP27, which can translate a small viral peptide, vSP27, that activates NF-κB signaling, suppressing BmCPV infection [108]. *Bombyx mori* Nucleopolyhedrovirus (BmNPV) is a critically important virus in silkworms, causing a significant economic impact on the silk production industry [118]. BmNPV was found to express multiple circRNAs; of them, circRNA-000010 could encode a small viral peptide termed VSP39 that acts as a proviral agent, boosting BmNPV virus replication [119]. Additionally, gibel carp (*Carassius gibelio*) is a uniquely important globally cultured freshwater fish species [120]. Cyprinid herpesvirus 2 (CyHV-2), which causes gill hemorrhagic disease and severe mortalities to gibel carps, encodes circ-udg, which can promote CyHV-2 proliferation and propagation [121].

## 4. Practical Applications of Circular RNAs

circRNAs differ from linear RNAs in conformation, immunogenicity, and stability. In particular, circRNAs are arguably more stable and less immunogenic than are other types of linear RNAs. These characteristics render circRNAs superior to linear RNA for practical applications. Therefore, several efforts have been made to create circRNA-based formulas, including non-coding aptamers, antisense RNAs, templates for sustained translation, modulators of innate immune responses and miRNAs, diagnostic and prognostic biomarkers for viral infections, and vaccine candidates [19].

The immunogenicity of synthetic circRNAs must be assessed and, if necessary, tailored for optimal biomedical uses. Recently, self-circRNAs were demonstrated to be commonly programmed by introns and paired with RBPs, denoting their origins. Nonself-circRNAs are distinguishable, and RIG-I-mediated signaling triggers antiviral immune responses after sensing them [122]. Breuer et al. [123] demonstrated that synthetic circRNAs might be used as miRNA sponges if they are generated in a cell-free system via in vitro transcription and ligation and purified via gel extraction [123].

### 4.1. Vaccine Candidates

Being chemically stable and less immunogenic than linear mRNAs [42,124], it has been suggested that synthetic and adeno-associated viral-based translatable circular RNAs can be incorporated into circRNA-based therapies [42,125,126,127]. Additionally, lipid nanoparticles (LNPs) are employed to deliver circRNA vaccines and therapeutics [42,52]. In mouse adipose cells and tissues, the nano-formulated administration of unmodified IRES-containing circRNAs improved the length of translation duration relative to that of linear mRNAs [42,124]. Of note is that circRNA-LNPs demonstrated significantly greater thermostability than did linear mRNA-LNPs [52]. Unlike current mRNA vaccines, necessitating strict transportation and storage conditions [128], circRNA vaccines encapsulated in LNP can be effectively kept for four weeks at 4 °C and up to two weeks at room temperature [52].

Intriguingly, it was hypothesized that the circRNA vaccine could exploit the observation that non-self circRNAs can activate RIG-I and PKR signaling pathways to function as a self-adjuvant and further boost the immune response induced by vaccination [42,43,115]. Nevertheless, its immunogenicity must be optimized to maintain the desired adjuvant effect without inducing adverse reactions that could significantly reduce circRNA vaccine efficacy [129].

**Figure 2:**
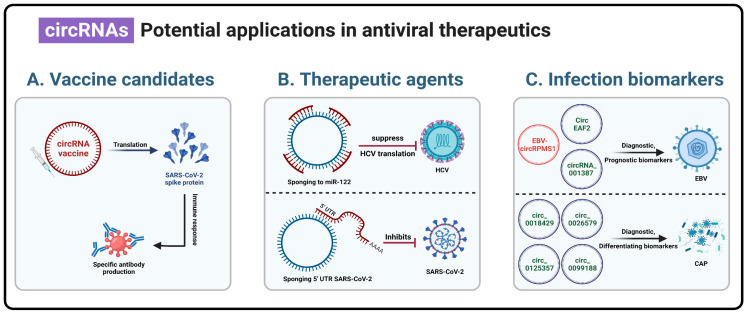
Potential applications of circRNAs in antiviral therapeutics. The relatively more stable and less immunogenic nature of circRNAs compared to that of their linear cognate RNAs renders circRNAs a superior arsenal for potential antiviral therapeutics and a new biomarker for the detection of strenuously diagnosed diseases caused by viruses. (**A**) Circular RNA vaccines expressing the RBD of the SARS-CoV-2 spike protein demonstrated neutralizing antibody generation in mice and monkeys [52,55,130]. (**B**) Top panel: a circular RNA sponge containing miRNA-122 binding sites, sequestered miRNA-122, and suppressed HCV viral replication [48]; Bottom panel: antisense circRNAs targeting distinct regions of the 5′-UTR of SARS-CoV-2 that can efficiently inhibit virus replication [132]. (**C**) Top panel: EBV-encoded circRPMS1 and host hsa_circRNA_001387 that are significantly expressed in EBV-positive NPC tissues. Hence, they could be valuable biomarkers for the diagnosis and prognosis of NPC [133,134]; CircEAF2 inhibited EBV and large B-cell lymphoma progression via the miR-BART19-3p/APC/β-catenin axis, indicating it is a potential prognostic biomarker [99]. Bottom panel: four circular RNAs (hsa_circ_0018429, hsa_circ_0026579, hsa_circ_0125357, and hsa_circ_0099188) which were shown to be very sensitive and specific biomarkers for the diagnosis of community-acquired pneumonia (CAP) [135]. HCV: Hepatitis C virus; EBV: Epstein–Barr virus; CAP: community-acquired pneumonia. Created with BioRender.com (accessed on 4 August 2023).

circRNA vaccines expressing the receptor-binding domain (RBD) of the SARS-CoV-2 spike protein demonstrated sustained antigen production, cellular and humoral immune responses, neutralizing antibody formation in mice and monkeys, and distinct Th1-biased immune responses (Figure 2A) [52,55,130]. These recent findings substantiate the therapeutic benefits of circRNAs for sustained expression. Nevertheless, a number of crucial concerns must first be resolved, including IRES optimization and other strategies facilitating cap-independent translation [127,131], as well as reducing the cellular immune responses induced by the administration of the circRNA vaccine [43,55].

### 4.2. Therapeutic Agents

circRNAs can absorb miRNAs, reducing their bioaccessibility and activity against their intended mRNA targets. This led to the introduction of ectopically produced or in vitro-generated circular RNAs expressing partial MREs sites, enabling a reduction in disease- or virus-related miRNA activity in vitro and in vivo [48,136], giving a potential therapeutics alternative to the existing gold standard, antagomirs [137].

The HCV functional sequestration of miRNA-122 in cells is an excellent example of this approach. miRNA-122 is critical for HCV replication and propagation as it binds to the HCV RNA 5′-end, stabilizing and protecting the HCV genome from nucleolytic degradation and boosting HCV viral replication. Miravirsen, a locked nucleic acid (LNA)-modified DNA phosphorothioate antisense oligonucleotide complementary to miRNA-122, currently undergoing clinical trials, disrupts miRNA-122’s protective role on HCV RNA [138]. Briefly, 5′-3′-end-ligation using T4 RNA ligase 1 was used to generate a circular RNA sponge containing four miRNA-122 binding sites, thus sequestering miRNA-122 and suppressing HCV viral protein synthesis more effectively than did Miravirsen (Figure 2B) [48].

Additionally, SARS-CoV-2 viral replication was significantly suppressed in a cell culture by a panel of antisense circular RNAs that were designed to target the structurally conserved 5′-UTR of the virus’s genomic RNA [132]. Antisense-circRNAs targeting distinct regions of the 5′-UTR of SARS-CoV-2 efficiently inhibited virus replication by up to 90% compared to the control, and the durability was at least 48 h (Figure 2B) [132].

Additionally, two interesting Chinese herbs (*Oldenlandia diffusa* (Willd.) and *Scutellaria barbata* D.Don (SB)) were shown to dramatically reduce HBV activity, and HCC growth, migration, and invasion both in vitro and in vivo [139]. This activity may have been due to the modulation of the circRNA–miRNA–gene expression network [139].

### 4.3. Viral Infection Biomarker

circRNAs are detected as enriched detectable components in exosomes [140,141] and as a component of physiological body fluids (such as saliva, blood, and urine); they are also prevalent in peripheral tissues [142,143,144,145,146]. These properties make circRNAs stable and resistant to environmental fluctuations, rendering them potential biomarkers for detecting various diseases and infections [147].

For instance, diagnosing NPC, a disease typically caused by EBV, can be challenging; consequently, early detection can benefit therapeutic management. In this context, host hsa_circRNA_001387 and EBV-encoded circRPMS1 are significantly expressed in EBV-positive NPC samples. Hence, EBV-circRPMS1 and cellular hsa_circRNA_001387 could be valuable biomarkers for the diagnosis and prognosis of NPC [133,134]. Additionally, it was reported that the overexpression of circEAF2 in EBV-positive B lymphoma cells induces cell apoptosis and sensitizes lymphoma cells to epirubicin. circEAF2’s preferential target is the EBV-encoded miR-BART19-3p, which upregulates the tumor suppressor adenomatous polyposis coli (APC) and inhibits downstream β-catenin production, leading to the inactivation of the Wnt signaling pathway and suppression of EBV and DLBCL cell proliferation. CircEAF2 impacted the miR-BART19-3p/APC/β-catenin axis, and consequently inhibited EBV and large B-cell lymphoma progression, indicating that it is a potential prognostic biomarker (Figure 2C) [99].

Recently, four circRNAs (hsa_circ_0026579, hsa_circ_0018429, hsa_circ_0099188, and hsa_circ_0125357) were shown to be very sensitive and specific biomarkers for diagnosing community-acquired pneumonia (CAP). Interestingly, hsa_circ_0026579 was proposed as a circRNA biomarker that can distinguish the causative agent of CAP to be either viral/bacterial or mixed infection (Figure 2C) [135]. In addition, He et al. [148] reported that patients with dengue fever showed considerable upregulation of hsa_circ_0015962 and significant downregulation of hsa_circ_0006459. The upregulation of hsa_circ_0015962 and downregulation of hsa_circ_0006459 influence the therapeutic response to dengue fever and are promising biomarkers in dengue fever patients [148].

## 5. Summary

The biological functions of circRNAs have drawn the scientific community’s attention in the past years. Importantly, recent research has revealed that circRNAs could play a role in inducing or dampening antiviral immunity. It has been shown that circRNAs potentially regulate the expression of genes implicated in innate immunity, serving as either antiviral or proviral host factors. These findings contribute to the ever-growing comprehension of physiological and pathophysiological functions of such ncRNAs. However, our current knowledge and advancements in circRNA research are limited. For instance, the mammalian transcriptome contains a vast number of circRNAs with unknown functions that remain to be determined. Moreover, during circRNA synthesis, the backsplicing of pre-mRNA is frequently accompanied by alternative splicing. How the splicing machinery decides and chooses between RNA splicing, alternative splicing, and backsplicing to generate circRNAs is poorly understood. Consequently, it is essential to have a thorough understanding of the processes underlying circRNA synthesis and degradation.

Although progress has been made in the understanding of circRNAs’ involvement in the innate immune response to viral infection, the exact mechanism of how they regulate innate immunity is still unclear. Extensive studies of circRNA expression and function in response to viral infections may provide a solid basis for a better understanding of regulatory networks that protein-centric research might have underestimated. CircRNAs could potentially serve as proper biomarkers for a number of infections and diseases, therapeutic agents, and vaccine candidates, but these need to be further investigated. One of the challenges confronting circRNAs research is the lack of a well-established in vivo system for depleting circRNAs of interest. Since circRNAs share the same pre-mRNA with their cognate mRNA, it is challenging to alter the expression of only circRNAs without influencing the expression of the linear mRNA. Limited success has been achieved in developing some specific circRNA-depleted animal models. However, establishing a methodology to knock out merely circRNA expression effectively is an ongoing task. The development of such an in vivo system would be a breakthrough in investigating the biogenesis and functions of circRNAs in vivo.

## Figures and Tables

**Table 1:** List of reported virus-dysregulated circRNAs and their functions/mechanisms in regulating innate immunity or viral replication.

circRNA	Stimuli	Differential Expression	Functions/Mechanisms	Reference
hsa_circ_0001400	KSHV	Up	During KSHV de novo infections, circ_0001400 expression suppressed the expression of vital latent and lytic viral genes without significantly altering the viral genome copy number.	[89]
circ_0000479	HTNV	Up	Circ_0000479 sponged miR-149-5p and regulated RIG-I expression, thus dampening viral replication.	[63]
hsa_circ_0004812	HBV	Up	Circ_0004812 silencing enhanced the expression of IFN-α and β in HBV-infected Huh7 cells.	[90]
circBACH1	HBV	Up	CircBACH1 regulated HBV propagation through the miR-200a-3p/MAP3K2 pathway.	[91]
circ-ATP5H	HBV	Up	Circ-ATP5H boosted HBV replication by modulating the miR-138-5p/TNFAIP3 axis.	[92]
circFNDC3B	MERS-CoV	UP	The silencing of circFNDC3B and circCNOT1 significantly suppressed the MERS-CoV viral load and its target mRNA expression, modulating various biological pathways, including the MAPK and ubiquitination pathways.	[69]
circCNOT1				
hsa_circ_0004445	MERS-CoV	UP	The knockdown of hsa_circ_0004445 inhibited MERS-CoV replication.	[93]
hsa_circ_0000479	SARS-CoV-2	UP	SARS-CoV-2 could regulate IL-6 and RIG-I activity via hsa_circ_0000479/hsa-miR-149-5p/RIG-I, IL-6axis.	[64]
ssc_circ_009380 (circEZH2)	TGEV	Down	CircEZH2 promoted the activation of NF-κB via sponging miR-22.	[67]
circMerTK	IAV	Up	CircMerTK inhibited IFN-beta production and suppressed IFN signaling, thus boosting IAV replication.	[31]
circRNA AIVR	IAV	Up	circRNA AIVR inhibited IAV replication by predominantly absorbing miR-330-3p.	[59]
circRNA_0050463	IAV	Up	CircRNA_0050463 was found to sponge to miR-33b-5p and thereby enhanced IAV replication.	[94]
circ-GATAD2A	IAV	Up	Circ-GATAD2A promoted influenza virus multiplication by inhibiting VPS34-dependent autophagy in vitro	[95]
hsa_circ_0005870	IAV	UP	The overexpression of these three circRNAs inhibited AIV replication and proliferation, whereas silencing these circRNAs enhanced AIV multiplication.	[96]
hsa_circ_0006104				
hsa_circ_0009365				
circEXOSC	HCV	UP	Depleting circEXOSC in HCV-infected cells markedly reduced viral infectivity.	[97]
circTIAL			A significant reduction in HCV infectivity was observed after circTIAL silencing.	
circPSD3	HCV	UP	CircPSD3 promoted HCV RNA abundances at a post-translational level.	
	DENV	UP	CircPSD3 was found to reduce viral infectivity in Dengue virus-infected cells significantly.	
ciTRAN	HIV	UP	HIV-1-Vpr-induced ciTRAN-sequestered SRSF1 from the HIV viral transcriptional complex to enhance viral transcription.	[98]
circSIAE	CVB3	Down	CircSIAE suppressed CVB3 replication by targeting miR-331-3p and TAOK2.	[65]
CircEAF2	EBV	Down	CircEAF2 inhibited the replication of EBV and the progression of DLBCL via the miR-BART19-3p/APC/β-catenin axis.	[99]

KSHV: Kaposi sarcoma-associated herpesvirus; HTNV: Hantaan virus; HBV: Hepatitis B virus; MERS-CoV: Middle East respiratory syndrome coronavirus; TGEV: Transmissible Gastroenteritis Virus; IAV: Influenza A virus; HCV: Hepatitis C virus; DENV: Dengue virus; HIV: Human immunodeficiency virus; CVB3: Coxsackievirus B3; EBV: Epstein–Barr virus.

## Data Availability

Not applicable.

## Abbreviations

APC	Adenomatous polyposis coli
BmCPV	*Bombyx mori* cypovirus
BmNPV	*Bombyx mori* Nucleopolyhedrovirus
BSJ	Backsplice junction
Calu-3	Human lung adenocarcinoma cells
CAP	Community-acquired pneumonia
CCNK	Cyclin-K
ceRNAs	Competing endogenous RNAs
CHB	Chronic Hepatitis B
circRNAs	Circular RNAs
ciRNAs	Circular intronic RNAs
CLDN18	Claudin 18
COVID-19	Coronavirus Disease 2019
CREBBP	CREB-binding protein
CVB3	Coxsackievirus B3
CVB3	Coxsackievirus B3
CyHV-2	Cyprinid herpesvirus 2
DENV	Dengue virus
DLBCL	Diffuse Large B-Cell Lymphoma
EBV	Epstein–Barr virus.
ecircRNAs	Circular exonic RNAs
eiciRNAs	Exon–intron circRNAs
EVD	Ebola Virus Disease
G3BP1	GTPase-activating protein SH3 domain-binding protein 1
HBV	Hepatitis B virus
HCC	Hepatocellular carcinoma
HCV	Hepatitis C virus
HDV	Hepatitis D virus
HepG2	Human hepatoma cell line
HIV	Human immunodeficiency virus
HK2	Hexokinase 2
hnRNP C	Heterogeneous nuclear ribonucleoprotein C
HRSP12	Heat-responsive protein 12
HTNV	Hantaan virus
Huh7	Human hepatocarcinoma cell line
IAV	Influenza A virus
ICAM1	Intercellular adhesion molecule 1
IFN	Interferon
IL-15	Interleukin-15
IL-6	Interleukin-6
IPEC-J2	Intestinal porcine enterocytes cell line
IRF3	Interferon Regulatory Factor 3
JAK-STAT	Janus Kinase/Signal Transducer and Activator of Transcription
KSHV	Kaposi sarcoma-associated herpesvirus
LNA	Locked nucleic acid
lncRNAs	Long non-coding RNAs
LNP	Lipid-based nanoparticle
MAP3K2	Mitogen-Activated Protein Kinase Kinase Kinase 2
MAPK	Mitogen-activated protein kinase
MERS-CoV	Middle East respiratory syndrome coronavirus
MIMV	maize Iranian mosaic virus
miRNAs	MicroRNAs
mPTP	Mitochondrial permeability transition pore
MREs	MiRNA response elements
mRNAs	Messenger RNAs
ncRNAs	Non-coding RNAs
NF-κB	Nuclear factor kappa light chain enhancer of activated B cells
NOD	Nucleotide-binding oligomerization domain
NPC	Nasopharyngeal carcinoma
p53	Tumor protein P53
PD-L1	Programmed death ligand 1
PKR	Protein kinase R
RBD	Receptor binding domain
sncRNAs	Small non-coding RNAs
SRSF1	Serine And Arginine-Rich Splicing Factor 1
TAOK2	Thousand-And-One Kinase 2
TAP2	Transporter 2
TGEV	Transmissible Gastroenteritis Virus
TNFAIP3	TNF alpha-induced protein 3
TRAF6	TNF receptor associated factor 6
TRIM59	Tripartite Motif Containing 59
VPS34	Vacuolar protein sorting 34
vSP27	Viral small peptide 27
VSP39	Viral small peptide 39
YTHDF2	YTH N6-Methyladenosine RNA-Binding Protein F2
